# Microbiological findings in emergency department patients with sepsis identified by the Sepsis-3 criteria: a single-center prospective population-based cohort study

**DOI:** 10.1186/s12245-021-00360-x

**Published:** 2021-07-23

**Authors:** Signe Trille Sørensen, S. M. Osama Bin Abdullah, Rune Husås Sørensen, Ram Dessau, Niels Høiby, Finn Erland Nielsen

**Affiliations:** 1 Department of Emergency Medicine, Copenhagen University Hospital, Ebba Lunds Vej 40A, Entrance 67, 2400, Bispebjerg and Frederiksberg, Copenhagen, NV Denmark; 2 Department of Emergency Medicine, Slagelse Hospital, Slagelse, Denmark; 3 Department of Clinical Microbiology, Slagelse Hospital, Slagelse, Denmark; 4 Department of Clinical Microbiology, Copenhagen University Hospital, Rigshospitalet, Copenhagen, Denmark; 5 Institute of Immunology and Microbiology, University of Copenhagen, Copenhagen, Denmark; 6 Copenhagen Center for Translational Research, Copenhagen University Hospital, Bispebjerg, Frederiksberg Denmark

**Keywords:** Sepsis, SOFA, SIRS, Emergency department, Microbiology, Mortality

## Abstract

**Background:**

Studies comparing the microbiological profiles among sepsis patients identified with either Sequential Organ Failure Assessment (SOFA) score or systemic inflammatory response syndrome (SIRS) criteria are limited. The aim was to examine if there are differences in the microbiological findings among septic patients identified by Sepsis-3 criteria compared to patients identified by the previous sepsis criteria, SIRS, and without organ failure. A secondary purpose was to examine if we could identify microbiological characteristics with increased risk of 28-day mortality.

**Methods:**

Prospective cohort study of all adult (≥ 18 years) patients admitted with sepsis to the Emergency Department of Slagelse Hospital, Denmark from 1st October 2017 to 31st March 2018. Information regarding microbiological findings was obtained via linkage between a sepsis database and the local microbiological laboratory data system. Data regarding 28-day mortality were obtained from the Danish Civil Registration System. We used logistic regression to estimate the association between specific microbiological characteristics and 28-day mortality.

**Results:**

A total of 1616 patients were included; 466 (28.8%; 95% CI 26.6%-31.1%) met SOFA criteria, 398 (24.6%; 95% CI 22.5–26.8%) met SIRS criteria. A total of 127 patients (14.7%; 95% CI 12.4–17.2%) had at least one positive blood culture. SOFA patients had more often positive blood cultures compared to SIRS (13.9% vs. 9.5%; 95 CI on difference 0.1–8.7%). Likewise, Gram-positive bacteria (8.6% vs. 2.8%; 95 CI on difference 2.8–8.8%), infections of respiratory origin (64.8% vs. 57.3%; 95 CI on difference 1.0–14%), *Streptococcus pneumoniae* (3.2% vs. 1.0%; 95% CI on difference 0.3–4.1) and polymicrobial infections (2.6% vs. 0.3% 95 CI on difference 0.8–3.8%) were more common among SOFA patients. Polymicrobial infections (OR 3.70; 95% CI 1.02–13.40), *Staphylococcus aureus* (OR 6.30; 95% CI 1.33–29.80) and a pool of “other” microorganisms (OR 3.88; 95% CI 1.34–9.79) in blood cultures were independently associated with mortality.

**Conclusion:**

Patients identified with sepsis by SOFA score were more often blood culture-positive. Gram-positive pathogens, pulmonary tract infections, *Streptococcus pneum*o*niae*, and polymicrobial infections were also more common among SOFA patients. Polymicrobial infection, *Staphylococcus aureus*, and a group of other organisms were independently associated with an increased risk of death.

## Background

Sepsis is a life-threatening condition resulting from a dysregulated host response to infection caused by bacterial, viral, fungal, or parasitic pathogens [1]. Sepsis is associated with a high mortality rate depending on sepsis severity and the incidence is increasing worldwide [2].

In 1991, a consensus conference [3] sought to standardize sepsis diagnosis by defining sepsis as a combination of an infection and the systemic inflammatory syndrome (SIRS). Subsequently, several attempts were made to redefine sepsis, since SIRS had focus on an inflammatory response. The use of SIRS criteria as a prognostic tool for sepsis identification show inadequate specificity and sensitivity, and SIRS criteria requires laboratory testing [2]. In 2016, the Sepsis taskforce (Sepsis-3) redefined sepsis as an organ dysfunction caused by a dysregulated host response to infection, hereby introducing Sequential Organ Failure Assessment (SOFA) score in the identification of sepsis. In addition, Quick SOFA (qSOFA), a modified version of the SOFA scoring system, was implemented to assist bedside clinicians in rapidly identifying patients as being at risk of a serious outcome [2].

Common foci of bloodstream infections are lungs, abdomen, and urinary tract [4–6]. Sepsis-causing organisms are most often Gram-negative or Gram-positive bacteria. Polymicrobial infections have also been identified in septic patients [1]. *Enterococcus*, *Acinetobacter*, *Pseudomonas* species, and *Staphyloccocus aureus* are associated with higher mortality [6, 7]. Furthermore, infections originating from the pulmonary tract have the highest mortality [8].

By changing the sepsis definition from an inflammatory response with or without organ failure, to now defining sepsis as a life-threatening organ dysfunction, we may expect a different microbiology among sepsis patients defined by Sepsis-3 criteria. Aside from substudies of the PHANTASi trial [9], the literature on culture positivity in septic patients identified by the Sepsis-3 criteria is sparse. Therefore, we primarily found it of interest to examine if there are any differences in the microbiological findings among septic patients identified by Sepsis-3 criteria compared to septic patients identified by the previous sepsis criteria, SIRS, and without organ failure. A secondary purpose was to examine if we could identify microbiological characteristics with increased risk of 28-day mortality.

## Methods

### Study design and setting

This study is a secondary analysis of data from a previously published paper [10]. The study was a prospective observational cohort study of all adult (≥ 18 years) patients with infection, admitted to the ED of Slagelse Hospital, Denmark, between 1 October 2017 and 31 March 2018. The Danish health care system is tax-funded, allowing equal access for all residents. All patients suffering from out-of-hospital acute illness are admitted to regional EDs. Private hospitals account for less than 1% of hospital beds in Denmark and patients with acute illness and in need of hospitalization are not provided treatment in private hospitals [11].

On arrival to the ED, a standardized electronic triage form was completed on all patients. Information regarding chief complaints was obtained, alongside with a short clinical assessment including vital measurements: blood pressure, respiratory rate, heart rate, peripheral oxygen saturation, core temperature, and level of consciousness by use of the Glasgow Coma Scale (GCS). All data obtained were electronically registered in the medical records. If patients with infection met either two or more qSOFA criteria, two or more SIRS criteria, or a general clinical evaluation gave suspicion of sepsis without fulfilling the sepsis criteria, a standard treatment protocol was initiated: the patients were examined by a physician within 10 min, arterial blood gas was drawn for analysis and treatment with oxygen, intravenous (IV) fluids, and antibiotics were administered. Electrocardiograms, blood samples, and blood cultures were routinely obtained for analyses. Foci of the infection were specified by bacterial culturing of possibly infected tissues and body fluids. As required, other examinations were performed: X-ray, ultrasound, computed tomography, gynecological examinations, etc.

Patients requiring hospitalization for more than 48 h after initial treatment were transferred to a medical ward. Critically ill patients were transferred to the intensive care unit (ICU).

### Definitions

In this study, patients were identified with infection if treatment with intravenous antibiotics was initiated within 24 h after presentation to the ED, and if the administration of antibiotics continued for at least 48 h. The SOFA sepsis group was defined by a SOFA score of at least two from baseline. In addition, blood cultures had to be drawn and first dose of intravenous antibiotics administered. The SIRS group was defined as patients meeting at least two SIRS criteria, and with similar demands regarding blood cultures and treatment with antibiotics as in the SOFA group. Patients with sepsis according to both SOFA score and SIRS criteria are only included in the SOFA group, since we want to distinguish patients with uncomplicated infection from patients with organ failure.

The qSOFA score [2] and SIRS criteria [3] variables and definitions used in this study was in accordance with the original guidelines.

Polymicrobial infection was defined as infection involving more than one species of microorganisms.

### Calculation of SOFA score

SOFA was not a routine method to identify sepsis during the study period. In the present study a baseline SOFA score of 0 was registered for all patients without comorbidities. We have adjusted the baseline SOFA score for chronic diseases that could have impact on the baseline value of SOFA. Patients with chronic respiratory, kidney, or liver diseases according to the Charlson Comorbidity Classification (CCI) [12] were assigned a SOFA baseline value from 1 to 4 depending on the severity of the chronic disease. This assessment was based on a combination of information on the grade of chronicity (mild, moderate, or severe kidney and liver disease) from the CCI classification and the arrival creatinine and bilirubin values. The adjustment for chronic pulmonary disease was based on information on pulmonary disease according to the CCI classification, and if different grades of decreased arrival PaO2 values at the ED were deemed to be chronically reduced. Patients with known dementia were assigned a baseline sofa score of 1. The decisions regarding adjustments for chronic diseases were made by consensus between two authors (OBA, RHS).

### Inclusion and exclusion criteria

Inclusion and exclusion criteria of patients were the same as in the previously published paper [10]. Patients included in the present study suffered from infection and met either SOFA and/or SIRS criteria on admission to the ED. Furthermore, patients were treated with intravenous antibiotics and had at least one blood culture obtained.

### Data collection

Information on demographics, medical history including Charlson Comorbidity Index (CCI) [13], vital measurements on admission, laboratory, and other test results were obtained from the electronic triage forms and medical records as described previously [10].

From the Danish Civil Registration System (CRS), an administrative registry with daily updated information on vital status of Danish citizens, data regarding deaths were collected using the unique Danish 10-digit CPR number.

The collected data were registered in an electronic database. Data collection and entry of data were randomly controlled by the authors (OBA, RHS) [10].

### Microbiological data

Data regarding microbiological results were obtained from the laboratory information system at the Department of Clinical Microbiology, Slagelse Hospital. This includes all microbiological test results dispatched from the ED of Slagelse Hospital during the study period.

Three bottles of 8–10 mL of blood were obtained per blood culture, two aerobic and one anaerobic bottle. The recommendation was to obtain blood cultures before intravenous antibiotic administration in patients with sepsis.

Gram-status, number of detected pathogens and foci of infection have been summed up in tables for the SOFA sepsis group and the SIRS sepsis group, respectively. Occurrence of the specific causative pathogens *Escherichia coli*, *Klebsiella pneumoniae*, *Staphylococcus aureus*, *Enterobacter cloacae*, and *Streptococcus pneumoniae* were analyzed according to the sepsis groups*.* Less frequently detected pathogens are presented as a separate group “other”. Coagulase-negative staphylococci were classified as contaminants and not included in the analyses. Blood cultures without growth of any pathogens were classified as culture-negative.

The clinical database was linked with the database containing all microbiological data by use of the unique Danish personal registration number.

### Statistical analysis

Continuous data are presented as medians with interquartile ranges (IQR) assuming non-normality. We have compared groups by using differences within medians with 95% confidence intervals (CI) and exact differences of proportions with 95% CI on differences. Differences were assumed significant if the 95% CI for the median difference or the 95% CI for the difference of proportions did not include 0.

Baseline characteristics, source of infection, distribution of pathogens in blood cultures, antibiotic treatment, in-hospital and 28-day mortality were analyzed according to the sepsis criteria. We have also analyzed baseline characteristics according to results (positive vs. negative) of blood cultures. We have analyzed the association between microbiological findings (positive blood cultures vs. negative blood cultures, Gram staining results, specific pathogens, and number of pathogens in blood cultures) and mortality in unadjusted and adjusted logistic regression models with age, gender, CCI, and the total SOFA score on admission as adjustment variables. Statistical analyses were performed using STATA v.15.1.

## Results

### Patients

During the study period, a total of 2112 patients were treated with antibiotics during admission in the ED and evaluated for inclusion in the present study. A total of 496 patients were treated with per-oral antibiotics and were excluded leaving 1616 patients with median age of 72.9 years (IQR 60.6–82.3). A total of 466 (28.8%; 95% CI 26.6–31.1%) patients had sepsis according to the SOFA criteria and 398 (24.6%; 95% CI 22.5–26.8%) patients had sepsis according to the SIRS criteria.

### Baseline characteristics

The baseline characteristics according to sepsis criteria are shown in Table 1. Patients in the SOFA group were older, the proportion of men was higher, the comorbidity burden was increased, and length of stay was longer. Systolic blood pressure, heart rate, peripheral oxygen saturation, GCS score, and core temperature were lower on admission to the ED (Table 1). Furthermore, patients in the SOFA group had higher values of creatinine, bilirubin, lactate, and glucose (Table 1). However, the white blood cell count and platelet count was lower in the SOFA group; the proportion of patients admitted to the ICU and receiving vasopressor therapy and ventilation therapy was higher among patients in the SOFA group (Table 1).

**Table 1:** Baseline characteristics among septic patients defined by SOFA or SIRS criteria

	SOFA(*n* = 466)	SIRS(*n* = 398)	Difference(95% CI)
Female sex, *n* (%)	195(41.9)	217(54.5)	12.6(6.0–19.2)
Age, median, years (IQR)	74.1(65–83)	71.2(56–80)	4.7(2.6–6.8)
Charlson Comorbidity Index, *n*			
0	109(23.4)	125(31.4)	8.0(2.0–14.0)
1–2	232(49.8)	195(49.0)	0.8(÷5.9–7.5)
3+	125(26.8)	78(19.6)	7.2(1.6–12.8)
Previously admitted with sepsis, *n* (%)	153(32.8)	110(27.6)	5.2(÷0.9–11.3)
Length of stay, median, days (IQR)	6.7(4.0–10.6)	4.3(2.7–7.0)	1.9(1.3–2.4)
Vital signs on admission, median (IQR)			
Severity of disease			
Total SOFA score	3(2–4)	0(0–1)	–
Systolic blood pressure, mmHg^a^ (IQR)	122(106–145)	135(122–150)	12(9–15)
≤ 90 mmHg, *n*(%)	37(7.9)	2 (0.5)	7.4(4.9–10.0)
Respiratory rate, breaths/min (IQR)	21(18–26)	21 (18–25)	0
Heart rate, beats/min (IQR)	95(81–112)	102(93–112)	7(4–10)
Peripheral oxygen saturation, %(IQR)	95(93–97)	96(94–98)	1(1–2)
Core temperature, °C^b^ (IQR)	37.7(36.8–38.5)	38.3(37.5–38.8)	0.5(0.3–0.7)
Glasgow Coma Scale < 15, *n* (%)	122(30.4)	30(7.5)	22.9(18.0–27.8)
Laboratory variables, median (IQR)			
C-reactive protein (mg/L)	94(36–167)	104(35–174)	3(÷13–7)
White blood cell count, (× 10^9^/L)	12.4(9.0–17.5)	13.8(10.5–17.3)	1.2(0.5–2.0)
Creatinine (μmol/L)	111(73–168)	74(61–92)	32(25–39)
Bilirubin (μmol/L)	11(7–17)	9(6–13)	2(1–2)
Platelet count (× 10^9^/L)	206(144–280)	256(207–324)	57(45–70)
Lactate (mmol/L)^c^	1.4(0.9–2.2)	1.0(0.7–1.7)	0.3(0.2–0.4)
Glucose (mmol/L)	7.3(6.1–8.9)	6.7(6.0–8.1)	0.4(0.1–0.6)
Admission to ICU, n (%)	73(15.7)	20(5.0)	10.7(6.7–14.6)
Vasopressor therapy, n (%)	14(3.0)	3(0.8)	2.2(0.4–4.0)
Ventilation therapy, n (%)	28(6.0)	7(1.8)	4.2(1.7–6.7)
Dialysis treatment, n (%)	3(0.6)	1(0.3)	0.3(÷0.5–1.2)

*CI* confidence interval, *ICU* intensive care unit, *IQR* interquartile range, *SOFA* Sequential Organ Failure Assessment, *SIRS* systemic inflammatory response syndrome,

^a^9 missing (7 and 2 in the SOFA and SIRS group, respectively)

^b^17 missing (10 and 7 in the SOFA and SIRS group, respectively)

^c^405 missing (175 and 230 in the SOFA and SIRS group, respectively)

Sources of infection, according to sepsis criteria, are shown in Table 2. The most common sites of infection were the pulmonary system and the urinary tract. Pulmonary system and central nervous system infections were more frequent in the SOFA group (Table 2).

**Table 2:** Source of infection among septic patients identified by SOFA or SIRS criteria

	SOFA, *n* (%) *n* = 466	SIRS, *n* (%) *n* = 398	Difference (95% CI)
Pulmonary	302(64.8)	228(57.3)	7.5(1.0–14.0)
Urinary tract	88(18.9)	95(23.9)	5.0(÷0.5 –10.5)
Gastro-intestinal	34(7.3)	44(11.1)	3.8(÷0.1–7.7)
Central nervous system	5(1.1)	0	1.1(0.1–2.1)
Skin	27(5.8)	29(7.8)	2.0(÷1.4–5.4)
Endocarditis	3(0.6)	1(0.3)	0.3(÷0.6–1.2)

*CI* confidence interval, *SOFA* Sequential Organ Failure Assessment, *SIRS* systemic inflammatory response syndrome

### Baseline characteristics according to culture-negative and culture-positive sepsis

A total of 127 (14.7%; 95% CI 12.4–17.2%) patients had at least one positive blood culture with isolated pathogens.

Patients with culture-positive sepsis had more often systolic blood pressures less than 90 mmHg, a total SOFA score > 3, and GCS< 15 on admission (Table 3). Heart rate, core temperature, C-reactive protein (CRP) values on admission, and length of stay (LOS) were increased compared to culture-negative sepsis (Table 3). Infection originating from the pulmonary system was more common in the culture-negative group whereas urinary tract infection was more frequently the source of infection in the culture-positive group (Table 3). Infections originating from the gastrointestinal tract, central nervous system, skin, or endocardium were equally distributed between the two groups (Table 3).

**Table 3:** Baseline characteristics according to blood culture results

	Culture-negative *n* = 737	Culture-positive *n* = 127	Difference (95% CI)
Sex			
Female	343(46.5)	69(54.3)	7.8(÷1.6–17.2)
Male	394(53.5)	58(45.7)	
Age, median (IQR)	72.9(59.9–81.1)	73.4(64.3–82.8)	1.9(÷4.6–0.7)
Charlson Comorbidity Index, *n*(%)			
0	197(26.7)	37(29.1)	2.4(÷6.1–10.9)
1–2	373(50.6)	54(42.5)	8.1(÷1.2–17.4)
3+	167(22.7)	36(28.4)	5.7(÷2.7–14.1)
Severity of disease			
SOFA, median (IQR)	2(0–3)	2(1–4)	0
SOFA > 3, *n*(%)	109(14.8)	37(29.1)	14.3(6.0–22.6)
SBP ≤ 90 mmHg, *n*(%)^a^	25(3.4)	14(11.0)	7.6(2.0–13.2)
RR, breath/min (IQR)	21(18–25)	22(19–29)	0(0–3)
HR, beats/min (IQR)	98(88–111)	106(93–118)	7(3–11)
Saturation, % (IQR)	96(94–98)	96(93–97)	0
Temperature, ^o^C (IQR)^b^	37.9(37.0–38.6)	38.4(37.7–39.1)	0.5(0.3–0.7)
GCS < 15, *n*(%)	137(18.6)	35(27.6)	9.0(0.7–17.3)
SIRS score, median (IQR)	2(2–3)	3(2–4)	1(÷1–0)
LOS, days, median (IQR)	5.0(31–8.4)	7.1(4.6–12.2)	2.0(1.1–2.9)
Laboratory variables, median (IQR)			
CRP	92(34–161)	141(68–249)	44(19.1–68.0)
WBC	13(9.6–17.1)	14.7(9.9–19.1)	1.1(÷2.5-0.3)
Focus of infection			
Pulmonary, *n*(%)	463(62.8)	67(52.8)	10.0(0.6–19.4)
Urinary tract, *n*(%)	139(18.9)	44(34.7)	15.8(7.1–24.5)
Abdominal, *n*(%)	67(9.1)	11(8.7)	0.4(÷4.9–5.7)
CNS, *n*(%)	4(0.5)	1(0.8)	0.3(÷1.3–1.9)
Skin, *n*(%)	46(6.2)	10(7.9)	1.7(÷3.3–6.7)
Endocarditis, *n*(%)	3(0.4)	1(0.8)	0.4(÷1.2–2.0)
I.V. AB treatment prior to BC, *n*(%)	115(15.6)	10(7.9)	7.7(2.3–13.1)
Time to first BC obtained, median hours (IQR)	1.4(0.8–3.7)	0.9(0.6–1.5)	0.4(0.3–0.6)
Number of BCs obtained, *n*(%)			
1	737(100)	106(83.5)	16.5(10.0–23.0)
2	0	12(9.4)	
3	0	7(5.5)	
4	0	1(0.8)	
5	0	1(0.8)	
> 1	0	21(16.5)	16.5(10.0–23.0)

*AB* antibiotic, *BC* blood culture, *CI* confidence interval, *CNS* central nervous system, *CRP* C-reactive protein, *GCS* Glasgow Coma Scale score, *HR* heart rate, *IQR* interquartile range, *IV* intravenous, *LOS* length of stay, *RR* respiratory rate, *SIRS* systemic inflammatory response syndrome, *SOFA* Sequential Organ Failure Assessment score, *SBP* systolic blood pressure, *WBC* white blood cells

^a^9 missing

^b^17 missing

The following variables were without significant differences between culture-positive and culture-negative patients: sex, age, CCI scores, number of SIRS criteria, and white blood cell count.

A total of 115 (15.6%) of the culture-negative patients received IV antibiotic treatment before blood cultures were drawn compared to 10 (7.9%) of the culture-positive patients (Table 3). Culture-positive patients had blood drawn for culturing earlier than culture-negative patients (Table 3).

### Blood cultures among septic patients identified by SOFA or SIRS criteria

The blood cultures of patients in the SOFA group were more often positive. Gram-positive bacteria were more frequently isolated from their blood cultures and detection of more than one pathogen in the blood cultures was also more frequent compared to the SIRS patients (Table 4). The total number of blood cultures with Gram-positive and Gram-negative bacteria was 51(40.2%; 95% CI 31.6–49.2%) and 64(50.4%; 95% CI 41.4–59.4%), respectively (Table 4).

**Table 4:** Blood culture characteristics and specific causative pathogens among septic patients identified by SOFA or SIRS criteria

	SOFA (*n* = 466)	SIRS (*n* = 398)	Difference (95% CI)
Time to BC, h, median(IQR)^a^	1.4(0.7–3.8)	1.2(0.7–2.7)	0.11(÷0.03–0.22)
Time to AB, h, median(IQR)^b^	4.3(2.6–7.5)	4.2(2.6–7.5)	0.1(÷0.5–0.4)
Treated with AB before BC	76(16.3)	49(12.3)	4.0(÷0.7–8.7)
Positive BC, *n* (%)^c^	65(13.9)	38(9.5)	4.4(0.1–8.7)
Gram-positive bacteria, *n* (%)	40(8.6)	11(2.8)	5.8(2.8–8.8)
Gram-negative bacteria, *n* (%)	37(7.7)	27(6.8)	0.9(÷2.5–4.4)
Polymicrobial infection, *n* (%)	12(2.6)	1(0.3)	2.3(0.8–3.8)
*Escherichia coli*, *n* (%)	21(4.5)	20(5.0)	0.5(÷2.4–3.4)
*Klebsiella pneumoniae*, *n* (%)	7(1.5)	2(0.5)	1.0(÷0.3–2.3)
*Staphylococcus aureus*, *n* (%)	7(1.5)	1(0.3)	1.2(÷0.02–2.40)
*Streptococcus pneumoniae*, *n* (%)	15(3.2)	4(1.0)	2.2(0.3–4.1)
Other^d^	22(4.7)	11(2.8)	1.9(÷0.6–4.4)
*Enterobacter cloacae*, *n* (%)	6(1.3)	1(0.25)	1.05(÷0.1–2.2)
Fungal infections, *n* (%)	1(0.2)	0	0.2(÷0.2–0.6)

*AB* antibiotic treatment, *BC* blood culture, *CI* confidence interval, *IQR* interquartile range, *SOFA* Sequential Organ Failure Assessment, *SIRS* systemic inflammatory response syndrome

^a^Time from admission to first blood culture obtained

^b^22 missing time to AB (13 and 9 for SOFA and SIRS, respectively)

^c^At least one positive blood culture, and coagulase negative staphylococci excluded

^d^Other: *Acinetobacter lwoffii*, *Actinotignum schaalii*, *Aerococcus urinae*, *Bacteroides theraiotaomicron*, *Bacteroides vulgatus*, *Candida albicans*, *Citrobacter freundii*, *Clostridium ramosum*, *Enterobacter cloacae*, *Enterococcus casseliflavus*, *Enterococcus faecalis*, *Enterococcus faecium*, *Gemella morbillorum*, *Hemolytic streptococci*, *Klebsiella oxytoca*, *Micrococcus species*, *Proteus mirabilis*, *Proteus vulgaris*, *Pseudomonas aeruginosa*, *Serratia marcecens*, *Streptococcus anginosus*, *Streptococcus mitis*, *Streptococcus mutans*

Time to blood culture obtained, time to administration of antibiotics, number of patients treated with antibiotics before the blood cultures were obtained, and number of patients with Gram-negative blood cultures were not different according to sepsis criteria (Table 4). *Streptococcus pneumonia* was more frequently detected in the SOFA group (Table 4). Number of patients with *Escherichia coli*, *Klebsiella pneumoniae*, *Staphylococcus aureus*, *Enterobacter cloacae*, other pathogens, and *Fungi* did not differ between SOFA and SIRS patients (Table 4).

### Mortality

Twenty-eight-day and in-hospital mortality was higher among patients fulfilling the SOFA criteria for sepsis (Table 5). The 28-day mortality in the SOFA and SIRS group was 14.4% (95% CI 11.3–17.9%) and 4.8% (95% CI 2.9–7.4%) (95% CI on difference 5.8–13.4%). In-hospital mortality was 7.9% (95% CI 5.7–10.8%) and 1.5% (95% CI 0.6–3.3%) (95% on difference CI 3.7–9.1%), respectively.

**Table 5:** Mortality among septic patients identified by SOFA or SIRS criteria

	SOFA, *n* (%, 95% CI) *n* = 466	SIRS, *n* (95% CI) *n* = 398	Difference (95% CI)
28-day mortality, *n* (%)	67(14.4; 11.3–17.9)	19(4.8; 2.9–7.4)	9.6(5.8–13.4)
In-hospital death, *n* (%)	37(7.9; 5.7–10.8)	6(1.5; 0.6–3.3)	6.4(3.7–9.1)

*CI* confidence interval, *SOFA* Sequential Organ Failure Assessment, *SIRS* systemic inflammatory response syndrome

### Microbiology variables and 28-day mortality

In an unadjusted regression analyses, we found that Gram-positive pathogens, polymicrobial infections, *Staphylococcus aureus*, and the group of other microorganisms in blood cultures increased the risk of mortality (Table 6). After adjustment for age, gender, CCI, and total SOFA score on admission, we found that polymicrobial infections in blood cultures (OR 3.70; 95% CI 1.02–13.40), *Staphylococcus aureus* (OR 6.30; 95% CI 1.33–29.80) and the pool of “other” microorganisms (OR 3.88; 95% CI 1.34–9.79) in blood cultures were independently associated with increased 28-day mortality (Table 6). Although the estimates were imprecise, the finding of *Escherichia coli* in blood cultures was associated with a decreased risk (OR 0.16; 95% CI 0.02–1.21) of death and Gram-positive bacteria increased the risk of death (OR 2.00, 95% CI 0.80–5.03) after adjustment.

**Table 6:** Crude and adjusted odds ratios for 28-day mortality

	Crude OR (95% CI)	Adjusted OR (95% CI)^a^
Blood cultures		
Negative	Reference	Reference
Positive	1.50(0.85–2.64)	1.16(0.63–2.15)
Gram staining		
Negative blood culture	Reference	Reference
Gram-negative	1.27(0.56–2.90)	1.03(0.43–2.49)
Gram-positive	2.58(1.14–5.83)	2.00(0.80–5.03)
Number of pathogens		
1	Reference	Reference
> 1	5.94(1.90–18.6)	3.70(1.02–13.40)
Pathogens		
*Escherichia coli*	0.22(0.03–1.60)	0.16(0.02–1.21)
*Klebsiella pneumoniae*	1.13(0.14–9.10)	0.79(0.09–6.82)
*Staphylococcus aureus*	9.43(2.31–38.44)	6.30(1.33–29.80)
*Streptococcus pneumoniae*	2.48(0.86–7.65)	2.16(0.60–7.77)
*Enterobacter* spp.	3.68(0.70–19.27)	2.08(0.32–14.30)
Other^b^	3.67(1.65–8.18)	3.88(1.34–9.79)

*CI* confidence interval, *OR* odds ratio

^a^Adjusted for age, gender, Charlson Comorbidity Index, SOFA score on admission

^b^The microorganisms are specified in footnotes in Table 4

## Discussion

This is the first study that has examined microbiological characteristics in septic ED patients identified by the new Sepsis-3 criteria and compared the results with uncomplicated septic patients defined by SIRS criteria. Patients fulfilling the SOFA criteria for sepsis were more often blood culture-positive and Gram-positive bacteria were more frequently isolated in the cultures. The SOFA patients suffered more frequently pneumonia. *Streptococcus pneumoniae* and more than one pathogen were more frequently isolated from their blood cultures. Polymicrobial infection, *Staphylococcus aureus*, and an unspecified group of other microorganisms in blood cultures were independently associated with 28-day mortality.

The 28-day mortality was significantly higher in the SOFA group compared to the SIRS group, which was to be expected since the SOFA group unlike the SIRS group consisted of septic patients with organ failure and generally appeared sicker on admission to the ED. The increased risk of complications and serious outcomes in septic patients with organ failure in general has, in our study, generated the hypothesis that SOFA patients may have more complex microbiological findings associated with serious outcomes.

Previous studies have shown an association between positive blood cultures in septic patients identified by SIRS criteria and number of organ systems affected, severity of disease, and mortality [9, 14]. Aside from an independent association between positive blood cultures and mortality in one of the studies [9], no specific microbiological findings were found independently associated with increased mortality.

Our study has demonstrated an independent association between microbiological characteristics including polymicrobial blood stream infections and mortality. The risk of death was in our study almost four times higher among patients with polymicrobial infection in their blood cultures. Similarly, it has previously been shown in a study of patients with clinical signs of sepsis identified by the SIRS criteria or with clinical indications of systemic infection, that patients with polymicrobial infections had a more than a two-fold risk of 90-day mortality [15]. These findings suggest that a more complex microbiological profile is associated with a worse prognosis.

The prevalence of Gram-positive bacteria was increased in the SOFA sepsis group. As a cause of sepsis, Gram-positive bacteria have increased in frequency over time, likely as a result of an increase in hospital-acquired infections and greater use of invasive procedures [1, 16]. Our finding of an increased number of patients with Gram-positive bacteria in a sepsis group, now defined by occurrence of organ failure, is in agreement with a study by Tulloch et al. [4]. They found a majority of Gram-positive isolates among patients identified with sepsis according to SIRS criteria and with 90% of the included patients having severe sepsis or septic shock. Although the estimate was imprecise, our results also suggest an increased risk of death among patients with Gram-positive pathogens in the blood cultures*.* In contrast to our finding, a meta-analysis of studies of patients with bloodstream infections showed that Gram-negative bacteraemia was associated with a higher mortality rate than infection with Gram-positive organisms [8]. Furthermore, they found *Escherichia coli* to be of lesser severity, our results support this finding.

Among specific microbes, we found that *Staphylococcus aureus* in blood cultures was independently associated with excess mortality. Unfortunately, we have no data in the present study on bacteria-related site of infection and resistance to antimicrobial agents. However, both methicillin-resistant and methicillin-sensitive *Staphylococcus aureus* have previously been shown to be independently associated with mortality in patients with bloodstream infections [7].

A large pool of several microorganisms was also independently associated with mortality in our study. The small number of positive blood cultures and the low prevalence of specific microorganisms in that group hinder further analyses of the impact of the group on mortality.

Besides our key-findings, we found a relatively large part (88%) of our blood cultures to be negative. In comparison, studies by Kumar et al. [17], Panday et al. [9], and Oliveira-Netto et al. [18] found 28–56% patients culture-negative among patients with sepsis or septic shock. A review performed by Angus et al. found blood cultures to be positive in only one third of severe sepsis cases [19].

Several factors can have led to this significantly higher amount of culture-negative results in our study. A total of 15.6% of the culture-negative patients received antibiotic treatment prior to drawing of blood cultures, this is known to increase the possibility of negative blood cultures [20, 21]. Studies have also shown that the amount of blood obtained for culturing has an influence on the number of false-negative and false-positive blood cultures. According to De Plato et al. [22], volume of blood is the most important parameter when detecting microorganisms in the bloodstream. They have recommended 30–40 ml of blood obtained in total, distributed into two aerobic and two anaerobic bottles. In our study, only two aerobic and one anaerobic bottle were used resulting in 24–30 ml blood obtained, in some cases, less than the recommended amount. This may partly explain the high rate of culture-negative sepsis in our patients.

### Implications

Our study provides new knowledge about the microbiological characteristics in sepsis patients identified by the updated Sepsis-3 criteria. This knowledge can be used in the stratification of sepsis patients and to identification of patients at high risk of death. Although the final results of microbiological analyses are not available during the early admission period new laboratory techniques for rapid microbiological testing are under development. In this light, our findings can be helpful in identifying high-risk patients needing early and specific antimicrobial intervention to prevent serious outcomes.

## Strengths and limitations

This study has several strengths. By use of a study design that ensured inclusion of all infected patients admitted to the ED during the study period and by use of national registries with complete follow-up for vital status, we have reduced the risk of selection bias. Our cohort includes patients from a uniform tax-supported health care system, which reduces the risk of referral bias. Further on, the identification of the sepsis patients was based on the updated Sepsis-3 criteria from 2016.

There are some limitations. First, the method used to calculate SOFA adjusted for comorbidities with potential impact on the SOFA calculations was not described in the protocol before study start, and the calculations have not been validated. Misclassification of patients with sepsis identified by the SOFA score can therefore not be excluded. Second, we have only used admission variables to calculate the SOFA score among patients with infections. Serial SOFA measurements may have identified more patients with clinical deterioration and fulfilling the sepsis criteria during the ED stay or after the transfer to a ward. Third, for inclusion in this study, the patients had to fulfil either the SOFA or SIRS criteria for sepsis, blood cultures should have been drawn, and intravenous antibiotics delivered. Patients fulfilling sepsis criteria and treated with intravenous antibiotics without having blood cultures obtained (143 SOFA patients and 162 SIRS patients) were not included. We have no data reporting why these patients did not have blood drawn for culturing. However, blood cultures from these patients could also have contributed with important information on microbiological diagnoses in sepsis groups and the risk of bias due to the exclusion of the patients cannot be ruled out. Fourth, although it is recommended to obtain blood cultures before treatment with antibiotics, a significant number of patients in our study were treated with antibiotics before blood cultures were drawn. Obtaining blood cultures after initiating antibiotic therapy is associated with a substantial loss of pathogen detection and reducing the chances to measure the true value of a culture (positive or negative) [20]. Fifth, the microbiological findings in our study should be interpreted in the light of the median age being almost 73 years which is higher compared to other studies we have discussed [4, 14, 18, 20] and Sepsis-3 studies in general [23–26]. It has been shown that bacteraemia is more common in older compared to younger patients, and that catheter-associated urinary tract infection and Gram-negative bacteria are more common in blood stream infections in patients older than 65 years [27, 28].

Sixth, a larger sample size could have resulted in more precise estimations and made it possible to stratify the analyses. Finally, this study was a single-center study, which may limit the generalizability of the study results.

## Conclusion

This study found that patients identified with sepsis according to the Sepsis-3 criteria, SOFA score, were more frequently blood culture-positive compared to patients identified with sepsis according to SIRS criteria. Gram-positive bacteria were predominant as the causative pathogens and pulmonary tract infection, *Streptoccocus pneumoniae*, and polymicrobial infections were more common among SOFA sepsis patients. Furthermore, we found that polymicrobial infections, *Staphylococcus aureus*, and an unspecified group of other microorganisms in blood cultures were independently associated with 28-day mortality.

## Data Availability

The datasets used and/or analyzed during the current study are available from the corresponding author on reasonable request.

## Abbreviations

AB: Antibiotics
BC: Blood culture
BSi: Blood stream infection
CCI: Charlson Comorbidity Index
CI: Confidence intervals
CNS: Central nervous system
CPR: Civil Person Register
CRP: C-reactive protein
CRS: The Danish Civil Registration System
ED: Emergency department
GCS: Glasgow Coma Scale
HR: Heart rate
ICU: Intensive care unit
IQR: Interquartile range
IV: Intravenous
OR: Odds ratio
qSOFA: Quick Sequential Organ Failure Assessment
RR: Respiratory rate
SIRS: Systemic inflammatory response syndrome
SOFA: Sequential Organ Failure Assessment
WBC: White blood cells
